# Transcanthal Canthopexy for Involutional Lower Eyelid Entropion Corrects Horizontal Laxity

**DOI:** 10.1155/2024/4694296

**Published:** 2024-02-13

**Authors:** Shinjiro Kono, Motohiro Kamei

**Affiliations:** Department of Ophthalmology, Aichi Medical University Hospital, Nagakute, Japan

## Abstract

In this prospective observational study, we aimed to examine improvements in horizontal laxity after lower eyelid retractor advancement and transcanthal canthopexy for involutional lower eyelid entropion. The study included 19 sides in 15 patients with involutional entropion who underwent transcanthal canthopexy with the advancement of the lower eyelid retractor. Using the pinch test, the distance from the lowest part of the corneal limbus to the eyelid margin was measured using callipers. All measurements were performed preoperatively and at postoperative 3 and 6 months. Using the pinch test, the distance from the lowest part of the corneal limbus to the lower eyelid margin was significantly shortened during each postoperative follow-up period. None of the included cases experienced recurrence. Our results indicated that transcanthal canthopexy could preserve postoperative horizontal tightness.

## 1. Introduction

Involutional lower eyelid entropion (involutional entropion) is the most common type of entropion, in which the lower eyelid turns inwardly [1]. Corneal abrasion caused by the cilia can cause ocular pain, irritation, itching, burning sensation, tearing, photophobia, conjunctival injection, discharge, and vision loss [1–3].

The main etiologic factors for the development of involutional entropion comprise the vertical and horizontal laxities of the lower eyelid that allow the preseptal orbicularis oculi muscle (OOM) to move upward over the pretarsal OOM [1]. The lower eyelid retractor (LER) is advanced to address vertical laxity, while horizontal laxity is corrected by horizontal tightening or shortening of the lower eyelid, such as with the lateral tarsal strip (LTS) procedure, transcanthal canthopexy (TCC), or wedge resection [1].

Previous reports have shown surgical success after combination surgery of the posterior layer advancement of the LER (LER advancement) with an LTS procedure or TCC. This was achieved with vertical and horizontal laxities [4–6] (LER advancement) based on the double-layered anatomy of the LER, where the posterior layer is the main vertical traction component of the lower eyelid [7, 8]. Definite advancement and fixation of the posterior layer can securely correct the vertical laxity [4].

The LTS and TCC tighten the lower eyelid horizontally at the lateral canthus [5, 6]. The surgical time of the LTS was long, and the LTS occasionally caused a postoperative lateral canthal deformity [9]. To address the above two drawbacks of the LTS, transcanthal canthopexy was used to correct horizontal laxity instead of the LTS. Transcanthal canthopexy is an eyelid-tightening procedure at the lateral canthus, which involves using a suture, without the risk of postoperative lateral canthal deformity [10]. However, the horizontal tension generally weakens after a certain postoperative period [10].

Although a previous study has shown that the combination of the TCC and LER did not cause recurrence, none of the studies have quantified horizontal laxity after TCC [4–6]. Therefore, in this study, we performed the pinch test before and after the combined LER advancement and TCC procedure and evaluated the change in horizontal laxity of the lower eyelid.

## 2. Materials and Methods

### 2.1. Ethics Approval

The institutional review board (IRB) of Aichi Medical University Hospital approved this study, which was conducted in accordance with the tenets of the Declaration of Helsinki and its later amendments (approval number, 2023-093). The IRB granted a waiver of informed consent for this study based on the ethical guidelines for medical and health research involving human subjects established by the Japanese Ministry of Education, Culture, Sports, Science and Technology and the Ministry of Health, Labour and Welfare. A waiver was granted as the study was not interventional. Nevertheless, at the request of the IRB, an outline of the study was published on the Aichi Medical University website, which was available for public viewing and gave patients the option to refuse to participate in the study, although none did. Personal identifiers were removed from the records prior to data analysis.

### 2.2. Study Design and Patients

This prospective observational study included Japanese patients whose involutional lower eyelid entropion was corrected by one of the authors (SK) between October 2020 and December 2022.

Patients with a history of other eyelid or lacrimal surgeries were excluded. Of the 19 cases, all patients had no previous history of eyelid or lacrimal surgery.

### 2.3. Data Collection

Data on age, sex, affected side, and pinch test results were collected. The horizontal laxity of the lower eyelid was measured preoperatively, at postoperative 3 months, and at postoperative 6 months. Complete success was shown in corrected horizontal laxity.

**Figure 1:**
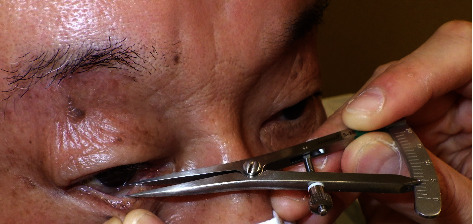
Measurement of horizontal laxity using the pinch test.

Horizontal laxity of the lower eyelid was measured using the pinch test. Prior to measurement, oxybuprocaine hydrochloride (0.4%) was ophthalmosed into the patient and left to rest for one minute. Subsequently, after the stinging sensation of the eye drops had disappeared, we applied a calliper and started measuring the distance. The center of the lower eyelid was pinched and pulled to the front, and a calliper was used to measure the distance between the lower eyelid margin and the eyeball. To prevent the measurement site from shifting during each measurement, we measured the distance from the corneal limbus just below the center of the pupil to the lower eyelid margin (Figure 1).

### 2.4. Surgical Procedure

The details of each surgical procedure have been presented in previous studies by the Department of Oculoplastic, Orbital, and Lacrimal Surgery at Aichi Medical University [4–6]. In this study, a process was added immediately after the TCC to check for correct horizontal tightening.

### 2.5. LER Advancement

Under local anesthesia, the skin was incised 3 mm below the cilia. The layer under the OOM was dissected toward the cilia. The anterior layer of the LER on the tarsus was detached inferiorly until the lower margin of the tarsus was exposed. The posterior layer of the LER was detached from the conjunctiva. The orbital septum was incised transversely just below the junction between the anterior layer of the LER and the orbital septum. The OOM at the eyelid margin was slightly debulked to facilitate outward rotation of the eyelid margin. Then, a site 2 mm below the edge of the posterior layer was fixed to the lower edge of the tarsus using a 6-0 Asflex® (Kono Seisakusho Co., Ltd., Tokyo, Japan) suture with simultaneous advancement of the anterior layer to reinforce the posterior layer. Subsequently, we added two additional sutures and secured the pretarsal OOM and the lower edge of the tarsus at three points. Finally, the skin was sutured using 6-0 Asflex® sutures [6].

### 2.6. TCC

Under local anesthesia, a 6-mm skin incision was made along the lateral canthal rhytids, just anterior to the lateral orbital rim. A stab incision was made at the lower eyelid margin, just medial to the commissure. Needles with double-armed 5-0 Prolene® sutures were inserted into the stab incision, passed through the hard lateral retinaculum or periosteum, and removed from the skin incision area [6]. Following firm ligation of this suture, the pinch test was performed to pull the lower eyelid forward, and after confirming that the horizontal tightening was stronger than that before the surgery, the skin was closed with 6-0 Asflex® sutures.

### 2.7. Statistical Analyses

The patient age and other measurement results are expressed as the mean ± standard deviation. The distance from the lowest part of the corneal limbus to the eyelid margin in the pinch test was compared among the three measurement periods using Friedman's test and Bonferroni correction because some of the measurement results did not show normal distribution. All statistical analyses were performed using SPSS ver. 26 software (IBM Corp., Armonk, NY, USA). Statistical significance was set at *P* < 0.050.

We also performed a post hoc analysis of the validity of the sample size in this study. The effect size was calculated based on the mean values and standard deviations of the pinch test and *F*-statistic. The *α* error was set as 0.05, and the power of the test (=1 − *β* error) was calculated using G *∗* Power software version 3.1.9.7 (Heinrich-Heine-Universita¨t Du¨sseldorf, Du¨sseldorf, Germany).

## 3. Results

The demographic data and measurement results are presented in Table 1. This study included 19 sides of 15 patients, among which bilateral involutional entropion was observed in four patients. The pinch test results before surgery ranged between 6 and 11 mm. The LER and TCC were performed on all the patients. None of the patients had any disease affecting wound healing or took medications that could have affected the recovery of the surgical site. The power of the test (=1 − *β* error) was 1.000, indicating the validity of the sample size in this study.

The results of the statistical comparisons are presented in Tables 1 and 2. The distance from the limbus to the eyelid margin was shorter at the 3- and 6-month follow-ups compared with the preoperative period. Although, at the 6-month follow-up, the distance was slightly longer compared with the distance measured at the 3-month follow-up, it was still shorter than that measured preoperatively. The distances measured at the 3- and 6-month follow-ups were significantly shorter compared with those measured preoperatively (*P* < 0.001), whereas the distances measured at the 3- and 6-month follow-ups were not significantly different (*P*=0.583).

The patients with a preoperative pinch test result of 6–11 mm underwent surgery, and at 6 months postoperatively, 17 of the 19 patients demonstrated a pinch test result of 5 mm or less. The two cases with a pinch test result of ≥5 mm at 6 months postoperatively included one case with a preoperative pinch test result of 11 mm, which reached 7 mm at 6 months postoperatively, and another with a preoperative pinch test result of 7 mm, which reached 6 mm at 6 months postoperatively.

Involutional entropion was successfully treated; none of the patients experienced recurrence at the 6-month follow-up. Additionally, none of the patients showed apparent lower eyelid retraction or ectropion after surgery.

## 4. Discussion

This is the first study to evaluate the horizontal tightening effect of the lower eyelid during LER advancement combined with transcanthal canthopexy. All patients experienced a horizontal tightening effect 6 months postoperatively. Furthermore, there were no postoperative complications such as ectropion or postoperative lateral canthal deformity. Additionally, none of the patients exhibited recurrence of the lower eyelid entropion.

Transcanthal canthopexy is thought to cause less lateral scar formation than the LTS [6, 10] and is thereby considered to be more prone to postoperative regression. However, in this study, none of the patients regressed to preoperative pinch test values 6 months postoperatively. Surgery was performed in patients with preoperative pinch test results ranging between 6 and 11 mm. Seventeen of the 19 patients showed a pinch test result of 5 mm at 6 months postoperatively. Although two patients had a pinch test result of ≥5 mm at 6 months, horizontal laxity was better than that observed preoperatively. In all cases, the pinch test value did not increase from the preoperative value, suggesting that transcanthal canthopexy was effective in tightening the horizontal direction.

Although a previous report defined a positive pinch test as 8 mm [11], we suggest that surgery may be performed without necessarily limiting the indication for a positive pinch test because surgery with LER advancement combined with transcanthal canthopexy requires less time and is effective in preventing recurrence [5, 6]. Older patients often have both horizontal and vertical laxity, and even if only vertical laxity is treated, lower eyelid entropion may recur later because of horizontal laxity. However, if the preoperative pinch test result is <5 mm, the influence of horizontal laxity on lower eyelid entropion is considered minimal, and surgery with LER advancement alone may be acceptable. Therefore, indications for LER advancement surgery alone should be considered for relatively young patients.

In this study, the horizontal tightening of the eyelid tended to loosen slightly at 6 months postoperatively compared with the tightening effect at 3 months postoperatively. This trend is consistent with the results by Yokoyama et al., who examined the lateral shift in the position of the lacrimal punctum after horizontal eyelid-tightening surgery for lower eyelid entropion [12]. Their results indicated that the horizontal distance from the lower punctum to the medial canthus increased during the 3-month follow-up period, while at the 6-month follow-up, the distance decreased from the baseline measured at the 3-month follow-up but was still longer than that measured preoperatively. Since the scar gradually softens during the scar remodelling phase within several weeks to a few years [13, 14], this may be caused by mild improvement in cicatricial contracture in the lateral canthus. In our study, the pinch test values at 3 and 6 months postoperatively were not significantly different, suggesting that the improvement in scar contracture of the lateral canthus was limited, and the tightening effect of TCC might be long-lasting. In the future, by collecting similar pre- and postoperative data on the pinch test in LTS and wedge resection surgery cases, it may be possible to determine the tightening effect of the LTS and wedge resection and to decide whether to use the TCC or other surgeries [5, 6, 15].

Although the snap back test and the medial and lateral distraction tests are also used to evaluate horizontal eyelid laxity, the pinch test is the most quantitative. The snap back test, observing the lower eyelid return to its original position after being pulled downwards [16], is qualitative. The degree of laxity is difficult to quantify. The medial and lateral distraction tests look at the shift in the position of the lacrimal punctum. These tests evaluate the laxity of the median canthal tendon and lateral canthal tendon [17]. Several studies [17–20] have investigated the grading method of MCT laxity. The methods for measuring lacrimal punctum shift have been demonstrated [12, 20]. However, the pinch test can directly assess horizontal eyelid laxity and is superior to these tests in ease of measurement. The pinch test is almost identical to the lid distraction test, in which the lower eyelid is gently pulled forward and away from the eye [16]. In the authors' assessment, a distraction of more than 6 mm is a sign of significant tarsoligamentous laxity [16]. This assessment is consistent with ours.

This study has some limitations that warrant discussion. First, we only included Japanese patients; because there are known racial differences in the eyelid anatomy [21], these results may not be applicable to other races. Second, all measurements were performed by a single examiner, which may have affected the reliability of the results. Third, quantification of the results of the pinch test for other entropion surgeries may provide more information for this study. Fourth, a longer follow-up period is needed to compare the long-term outcomes of the two techniques with those of other techniques in the future.

## 5. Conclusions

Our findings showed evidence that LER advancement combined with transcanthal canthopexy could have a long-lasting horizontal tightening effect on the lower eyelid.

## Figures and Tables

**Table 1:** Demographic data and results of measurements and statistical comparison.

Patient number/sides	15/19	
Male/female	8/7	
Right/left	8/11	
Bilateral	4	
Patient age (years)	73.4 ± 11.6	
Previous eyelid or lacrimal surgery	0	
Recurrence after surgery	0	
Postoperative lateral canthal deformity	0	

Mean pinch test (mm)	Mean value	Minimum to maximum value

Preoperative	7.89 ± 1.37	6.0–11.0
Postoperative 3 months	3.92 ± 0.80	3.0–5.0
Postoperative 6 months	4.55 ± 0.98	3.0–7.0
*P* value	<0.001	

**Table 2:** Results of Bonferroni correction.

Pinch test	Postoperative 3 months	Postoperative 6 months
Preoperative	<0.001	<0.001
Postoperative 3 months	—	0.583

## Data Availability

Data supporting the results of this study are available from the corresponding author upon reasonable request.
